# Opportunistic and Location-Based Collaboration Architecture among Mobile Assets and Fixed Manufacturing Processes

**DOI:** 10.3390/s18082703

**Published:** 2018-08-17

**Authors:** Dae Han Wi, Hyo Jun Kwon, Jung Kwang Park, Soon Ju Kang, Jae Duck Lee

**Affiliations:** 1 School of Electronics Engineering, College of IT Engineering, Kyungpook National University, 80 Daehakro, Bukgu, Daegu 41566, Korea; dnleogks23@naver.com (D.H.W.); bluekgssk@gmail.com (H.J.K.); miff214@naver.com (J.K.P.); 2 Smart Distribution Research Center, Advanced Power Grid Division, Korea Electrotechnology Research Institute, Changwon 51543, Korea; jdlee@keri.re.kr

**Keywords:** smart factory, opportunistic computing, location-aware, mobile asset management, IoT

## Abstract

Research into integrating the concept of the internet of things (IoT) into smart factories has accelerated, leading to the emergence of various smart factory solutions. Most ideas, however, focus on the automation and integration of processes in factory, rather than organic cooperation among mobile assets (e.g., the workers and manufactured products) and fixed manufacturing equipment (e.g., press molds, computer numerical controls, painting). Additionally, it is difficult to apply smart factory and IoT designs to analog factories, because such a factory would require the integration of mobile assets and smart manufacturing processes. Thus, existing analog factories remain intact and smart factories are newly constructed. To overcome this disparity and to make analog factories compatible with smart technologies and IoT, we propose the opportunistic and location-based collaboration architecture (OLCA) platform, which allows for smart devices to be attached to workers, products, and facilities to enable the collaboration of location and event information in devices. Using this system, we can monitor workers’ positions and production processes in real-time to help prevent dangerous situations and better understand product movement. We evaluate the proposed OLCA platform’s performance while using a simple smart factory scenario, thus confirming its suitability.

## 1. Introduction

Due to the advance of technology, many industrial factories are becoming automated in various ways. Current factory automation techniques mainly use centralized control-based production automation systems (i.e., Industry 3.0). However, future factories (i.e., Industry 4.0) will be equipped with real-time communication technologies that are attached to products moving throughout the factory [1,2]. It will also provide devices for workers to wear as well as providing a smarter environment for manufacturing facilities [3,4,5,6]. Currently, these smart factory technologies focus on automating and integrating production processes rather than device collaboration, and they do not suggest a model that connects products, workers, and manufacturing equipment together [7,8]. 

In addition, it is difficult to apply these device-collaborative smart technologies to traditional analog factories, because they must integrate both the necessary smart mobile assets and the existing manufacturing processes. Therefore, companies typically invest tremendous capital to instead build a new factory that is fitting the smart factory requirements. Especially in the existing analog factory, the work process control is not automated by the data flow, but the manual operation by the human operator. This causes a number of cases where a human operator’s mistake is caused by a serious accident at the factory. Although the mistake may be trivial, such as a minor error in setting the work process, it may directly affect the quality of the product.

It is also very important to understand the work process history management, which can verify whether the product was produced in an appropriate work environment under proper working conditions in each work process. 

This work process history management can now manage the entire process operation history by the central computer, due to this reason, this method cannot be applied to existing analog factories or factories for production of small quantity of locally distributed multi-products. For all production products, information on environmental conditions, working conditions in each process, and transportation information among processes should be separately managed for each product tray. Once this concept is established, it will be an important link to the production and consumption chains. Many researches on smart factory solution have been actively being carried out [9,10]. However, many of these studies have yet to provide an accurate solution to the problems that are mentioned above.

To solve these problems, we propose an opportunistic and location-based collaboration architecture (OLCA) platform, which enables devices for not only existing manufacturing equipment but also for mobile assets, such as wearable band for workers and mobile tags for product transport trays, to collaborate with each other using location information as well as opportunistic events occurring inside the factory. The proposed OLCA platform, in this paper, provides the ability to collaborate based on real-time location and opportunity-based production facilities, workers, and products in the factory. Therefore, this OLCA platform can be used not only in advanced smart factories, but also in certain analog type factories.

In Section 2, we will discuss the existing research related to this paper. In Section 3, we lay out the conceptual model of OLCA and the specific design of the model. In Section 4, we mention the implementation detail and its results, and in Section 5, we will provide a qualification for the validity and utilization of the OLCA model before we conclude in Section 6.

## 2. Related Research

### 2.1. Industry 4.0 and Trend of Smart Factory Platforms Researches

Recently, the concept of Industry 4.0 has been introduced, and the common platform for smart factory is emphasized as the application field that is technically most suitable for characteristics of Cyber-Physical Systems (CPS). It is a system that integrates and controls the system of the physical entity in cyberspace. By integrating cloud services with internet of things (IoT) in this CPS, it is the foundation of the Smart Factory to control processes, monitor alarms and manage services without direct human control. Many researches and developments have been recently made on this common platform, and various solutions have been proposed [9,11,12]. However, these recent attempts are not far from the concept of the traditional integrated factory automation, which is supervised and managed by the central computer. The extended concept of integrated factory automation can not satisfy the new concept of Industry 4.0, and especially, it is impossible to innovate the traditional analog factory without integrated factory automation facility. Therefore, our research dismisses the concept of simple factory automation and tries to focus on the problems that arise among objects that exist in the factory in common, including manufacturing equipment, such as press, computer numerical control (CNC), etc., human workers, and production products. These objects in factory are classified into three; one is fixed service device, next is mobile device, and the third is wearable device for workers. The problem occurred in real-time collaboration among them will be a key topic of this study. Therefore, this problem is technically similar to the problem of real-time location-based service (RTLS) and mobile asset management service rather than factory automation. The relevant research analysis on this field will be mentioned in the following section.

### 2.2. Real-Time Location System (RTLS) and Location Anchor Hub (LAH)

**Figure 1:**
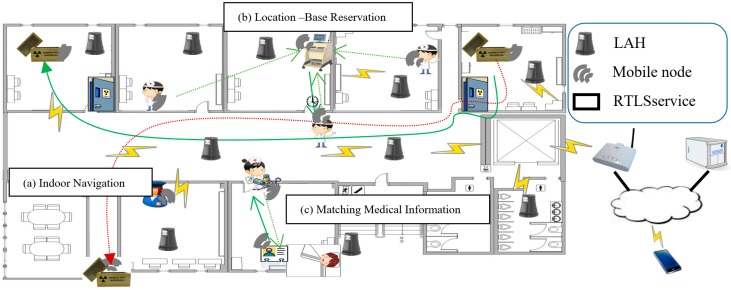
Location Anchor Hub (LAH) based real-time location system (RTLS) in a hospital.

This is a preliminary study of our research team before this study, and the LAH developed here is used in this study, so it is mentioned here to help the reader’s understanding. The LAH is an indoor location-based service hub with an overlay network. It is autonomously serviced without direct manipulation [13,14,15], and it recognizes the surrounding environment, providing services via autonomous collaboration among hubs without a central server. For example, in Figure 1b, the doctor requests location-based services, such as finding or scheduling a hospital equipment near the current location. The user is then provided with optimal service via the cooperation of hubs at each indoor space. The LAH has two roles in the RTLS application, the first is the role act as a location anchor for each mobile asset, and the second is the wireless communication access point in each unit space.

### 2.3. Proximity-Based Neighbor Identification Protocol (PNIP)

**Figure 2:**
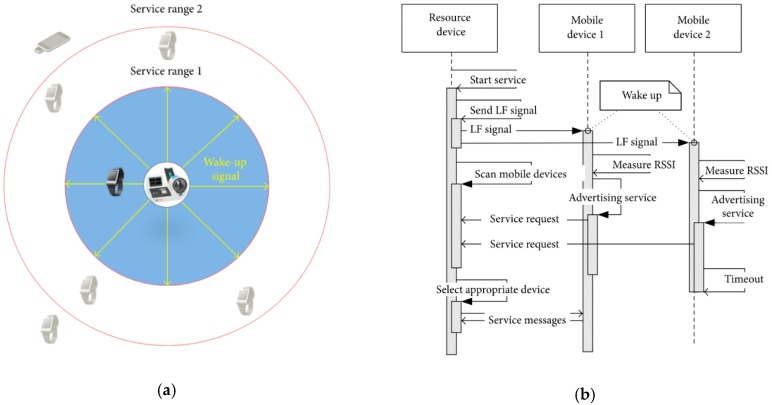
(**a**) Proximity-based neighbor identification protocol (PNIP) wakeup service; (**b**) Sequence diagram.

This is additional preliminary study of our research team before his study, and the PNIP is used in this study, so it is mentioned here to help the reader’s understanding. Proximity-based neighbor identification protocol (PNIP) is a hardware-based wakeup method that includes a software-based device-to-device (D2D) communication protocol for opportunistic services [16]. Figure 2a shows the PNIP wakeup service and Figure 2b shows the communication process of PNIP service with sequence diagram. The most important functions of PNIP are wirelessly waking the system from sleep mode and using low frequency (LF) (100–130 kHz) communications with high reliability. Such a wireless wakeup system using LF communication is also used in a vehicle smart key system [17]. When compared to the high frequency 2.4-GHz band, LF behaves better with obstacles, such as human bodies. The LF module generates and transmits a preamble to neighboring devices. One or more of those devices successfully receives the preamble and interrupts the main microcontroller unit (MCU). By utilizing an LF receiver chip that receives a predefined preamble, a wearable device awakens and exchanges data. Therefore, wearable devices can remain in sleep mode and facilitate ultralow power consumption [18]. Additionally, the response packet of the wearable device includes the identity and received signal strength (RSS) indicator (RSSI) value of the LF source device, enabling the accurate determination of distances between the LF source device and the wearable device. In this paper, we use PNIP to communicate between smart box and mobile devices.

### 2.4. Recent Related Researches for RTLS and Its Application in Smart Factory

Recently, many enterprises real-time locating system (RTLS) solutions using the 2.4-GHz band have been developed. Among them, a positioning technique that is based on Bluetooth low energy (BLE) RSSI has been widely used, because it operates with low power. However, it is difficult to attain a positioning accuracy of 1 m or less. In this study [19], they implement a 2.4-GHz industrial, scientific, and medical (ISM) radio frequency (RF) and ultra-wideband (UWB) hybrid radio frequency identification (RFID) real-time locating system combining UWB technology with existing 2.4-GHz RF. UWB provides cm-level location accuracy with time difference of arrival (TDoA)-based positioning technology [20]. Therefore, UWB is used where accurate position measurement is required, and 2.4-GHz RF is used where it is not. However, serious errors may occur in indoor environments where the time synchronization among the base stations is faulty or where there are problematic obstacles. Therefore, in a factory environment where obstacles are scattered, UWB RFID is not suitable. This requires an RTLS solution with LF that is less susceptible to obstacles. 

There has been study to recognize nearby devices while using Proximity Detection with RFID tag [21,22]. In this study, devices exchange data and locate nearby devices via a semi-passive RFID technology, complementing the traditional ultra-high frequency RFID system. This allows for better localization with simultaneous proximity detection among objects. However, Sense-a-Tags use a 900-MHz frequency. Obstacles (e.g., bodies and metal structures) can lower the RSS. When this happens, it is impossible to determine the exact distance between objects. Therefore, in a factory environment where various obstacles are scattered, RFID using 900-MHz frequency is not suitable. This problem can be solved with LF, which is less affected by obstacles and can measure more accurate distances. Additionally, the MCU must always operate in this scenario to receive the RF signal. Thus, there is a problem of high power consumption. However, by using PNIP, it is possible to realize not only more precise distances between objects, but also lower power consumption.

## 3. Concept of the OLCA Platform

### 3.1. Overview of the Proposed OLCA Platform

**Figure 3:**
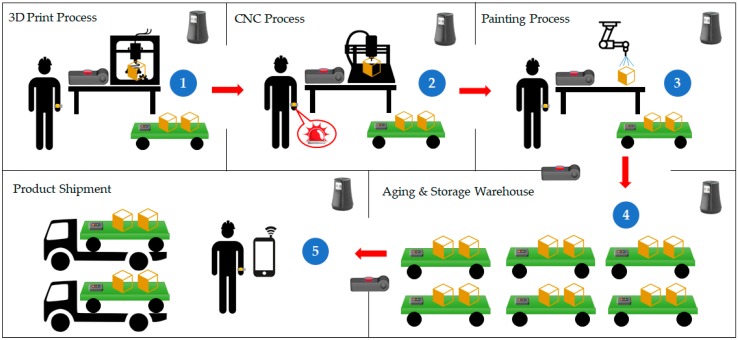
Opportunistic service scenario.

Figure 3 shows the service scenario of the proposed platform, describing the opportunities and location-based services of the factory. First, the worker designs the product and tests it while using a three-dimensional (3D) printer. Then, the completed design information is stored in the location-based anchor hub. Next, the worker uses a CNC machine to produce the product. The worker wears a smart band, a smart box is connected to the CNC machine, and a smart tag is installed on the cart carrying the product. When the worker presses the button on the smart box, it finds the nearest smart band and smart tag and it obtains the worker and product information. Then, via the LAH, the CNC machine fetches the product information stored in the previous step. If a problem occurs, the smart band informs the worker. When the product is completed, the smart tag stores the process information (i.e., worker information, process start time, process end time, trouble indicators, and surrounding environment information).

Next, the finished product is stored and treated (e.g., painted). Then, the time of entering and leaving the storage room is recorded in the smart tag. A smart box is installed at the doorway of the storage room to automatically detect the entry and exit of smart tags and smart bands. Additionally, if a product is shipped without sufficient storage time, it is possible to inform the worker and prevent potential product defects. Finally, the worker can check the production process information that is recorded in the smart tag with a smart phone, making it possible to know precisely which process failure has caused defects or other problems.

### 3.2. Requirements of OLCA Platform Design

The OLCA platform applies to smart factories. Communication that is less affected by object interference should be used. Therefore, LF communication should be used where accurate distance measurement is required. Elsewhere, it uses commonly used BLE and Wi-Fi communications. To provide opportunistic and location-based services while using RF and LF communications with multiple sensors, the following requirements must be met.

- Priority-based multitasking: to provide the services of this paper, devices must transmit and receive necessary information wirelessly with other devices in real time. Important time- or location-sensitive tasks should ensure stable operations via prioritization.
- Providing opportunistic services: occurs when a smart tag or smart band is located at a specific place via LF communications. Using the characteristics of the LF and the RSSI calculation, it is possible to measure the exact distance between devices, thereby determining the location and access status of the device.
- Providing location-based services: beacon-type messaging is required for location-based services. Devices periodically transmit their current position to the LAH using the BLE beacon. Currently, surrounding environment information is transmitted with data using various sensors.
- Low power operation of smart tag: because the smart tag is operated with a battery, it should not be turned off during operations. Low-power implementation is required, because environmental information is collected using sensors. In this paper, two MCUs are used to implement low power by independently controlling tag and sensor operations.

## 4. Detail Design and Implementation

### 4.1. Operation of the Opportunistic Service

**Figure 4:**
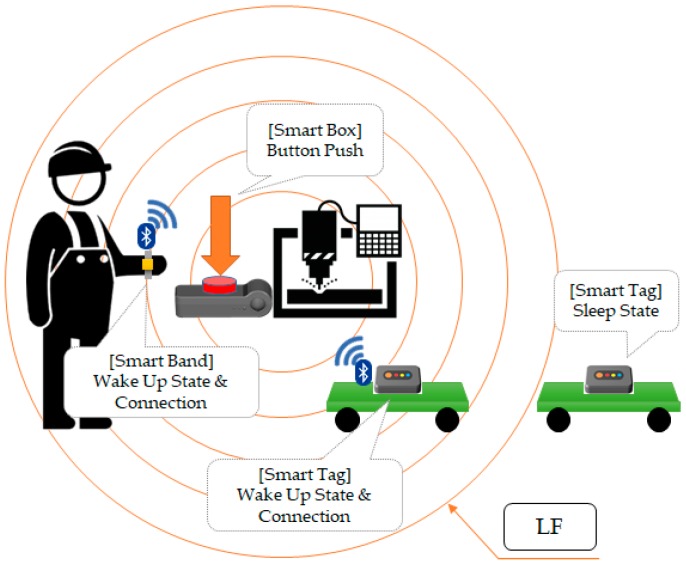
Concept of opportunistic service between mobile devices and manufacturing process.

Figure 4 shows the operation of opportunistic services. There are LF transmitters in the smart box and LF receivers in the mobile devices (i.e., smart band, smart tag), and they both communicate via BLE. When a worker initiates the smart box, its LF transmitter operates, and the mobile devices having an LF receiver wake up from sleep mode. Mobile devices that are not in the LF radius remain in sleep mode. The awakened mobile devices analyze the LF pattern and learn the smart box ID and RSSI information. When the mobile device advertises RSSI information and an ID, the smart box makes a connection with the nearest mobile device and shares its information.

**Figure 5:**
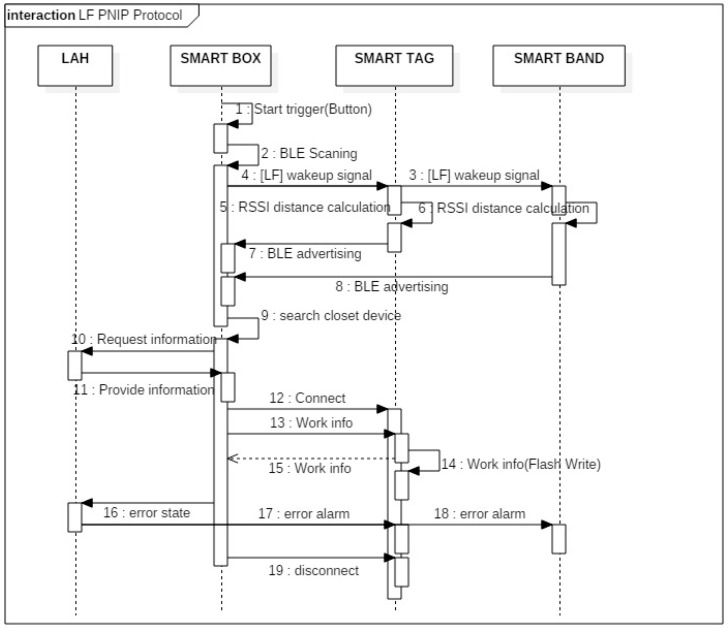
Communication between the LAH, smart box, smart tag, and smart band.

**Figure 6:**
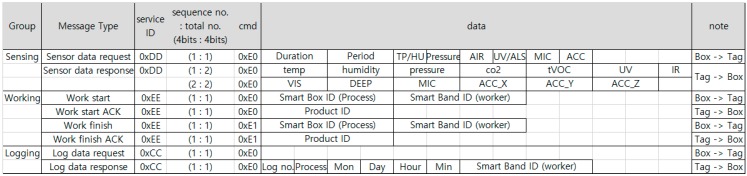
Communication protocol between the smart box and smart tag.

Figure 5 shows the sequence of the communication between the LAH, smart box and the mobile device when providing opportunistic services. Figure 6 shows the communication protocols that are defined for smart box and smart tag to transmit information to each other. When a smart box requests the required sensor information from a smart tag, the tag sends the corresponding sensor information in a packet. When starting or finishing a work, the smart box sends the process ID and worker ID to the smart tag, the tag records the working information in flash, and sends the product ID to the smart box. When the smart box requests the log from the smart tag, the tag sends the process, time, and worker information to the smart box.

### 4.2. Awareness of Neighbor Mobile Assets in Real-Time

**Figure 7:**
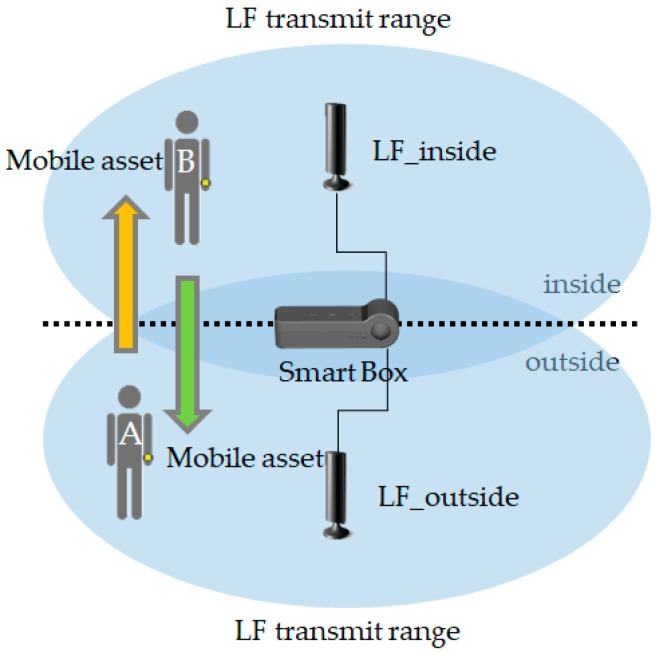
Identification of neighboring mobile assets.

Figure 7 shows the real-time mobile assets access detection method using opportunistic services. When mobile assets pass via the smart box, it determines whether the assets have entered or exited. A smart box with two LF transmitters is installed at the entrance and it periodically sends LF signals every 250 ms. The mobile device receives the inside and outside LF signals and determines whether the mobile asset has entered through the doorway using an RSSI calculation. This can also be used to determine if a worker is inside a hazardous area and can monitor the storage time of the product. It can even notify the worker when a product is shipped.

### 4.3. Unit Space-Based Precise Indoor Location Awareness

**Figure 8:**
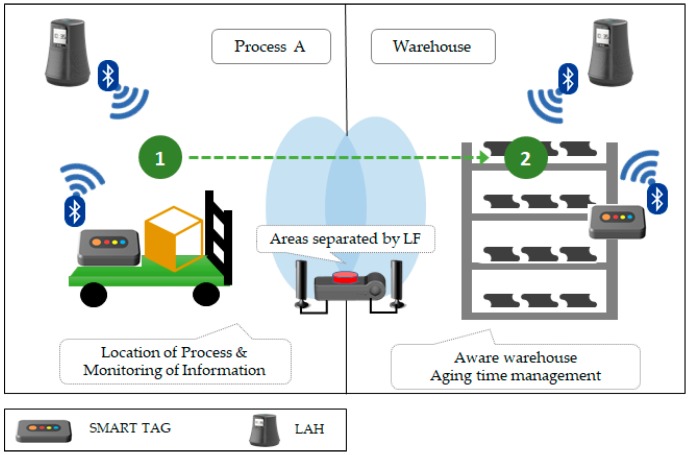
Indoor location awareness and service.

Figure 8 shows the indoor location and service. Mobile devices periodically perform BLE advertising, and LAH periodically performs BLE scanning. Thus, LAH recognizes in real-time which device is collaborating in its area. We also increase accuracy by adding an opportunistic service to the doorway. In Figure 8, the LAH in Process A recognizes the smart tag and monitors the environmental information. When the smart tag moves from 1 to 2, the LAH at each position determines that the smart tag has moved from Process A to the warehouse. The LAH that is installed in the warehouse checks the smart tag and manages the degree of product aging.

### 4.4. Multiple Sensor Control and Low Power Implementation

**Figure 9:**
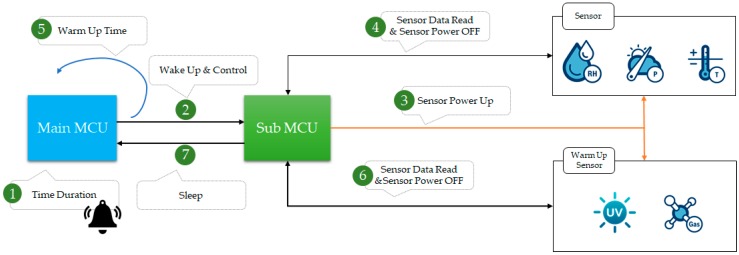
Multiple sensor control architecture of smart tag.

Figure 9 shows the low power implementation while using the main MCU and the sub-MCU with the smart tag. There are two ways to control multiple sensors. One is a method that always turns on the sensor, and the other is a method that turns it ON only when in use. The first method requires excessive power consumption and it is not suitable. There are two MCUs inside the smart tag to independently control it and the sensor. The sub-MCU turns on and reads data only when using the sensor. It receives control of the main MCU via inter-integrated circuitry communication and transmits sensing information. The general sensor can turn on the sensor when it reads the sensor data, but it must turn it on before reading the sensor data if it requires warming up.

## 5. Performance Evaluation

### 5.1. Opportunistic Service (CNC Process) Preparation Time

We implemented the CNC process scenario of Figure 3 while using the OLCA platform device that is proposed in this paper. When the button on the smart box was pressed, it searches for the nearest mobile device. the design information (i.e., Gcode) [23] was taken from the LAH using the mobile device’s information to operate the CNC machine. At this time, smart box brings design information from LAH through Wi-Fi communication. The period elapsed from pressing the button to the operation of the CNC machine was then measured. The size of the Gcode file used for the test was 2.54 KB. Table 1 shows the results obtained by repeating the following procedure 50 times. The average measurement result was 5.298 s and the standard deviation was 0.907 s.

### 5.2. Recognition of Multiple Mobile Devices Access

**Figure 10:**
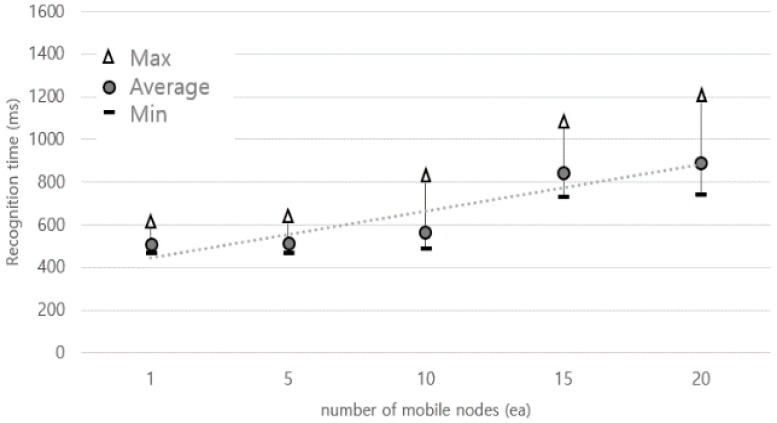
Recognition speed as mobile devices increase.

We implemented the mobile devices access service in Figure 7 while using OLCA platform devices and measured the recognition time when many mobile devices entered and exited simultaneously for performance evaluation. Although LF covers a very small distance of less than 5 m, basically, the LF-based locationing system guarantees a precision accuracy of 10 cm. The experiment in Figure 10 is an experiment that measures the time that it takes to recognize under the condition that all mobile devices recognize 100%. Figure 10 shows a graph of the time taken for all mobile devices to recognize per the number of mobile devices, as repeatedly measured 50 times. The X-axis represents the number of mobile devices, and the Y-axis represents the time taken for all mobile devices to be recognized. We confirmed that the time taken when 20 mobile devices entered and left at once was enough to respond to the movement speed of a moving person at the recognition range of 4 m within about 1 s.

**Figure 11:**
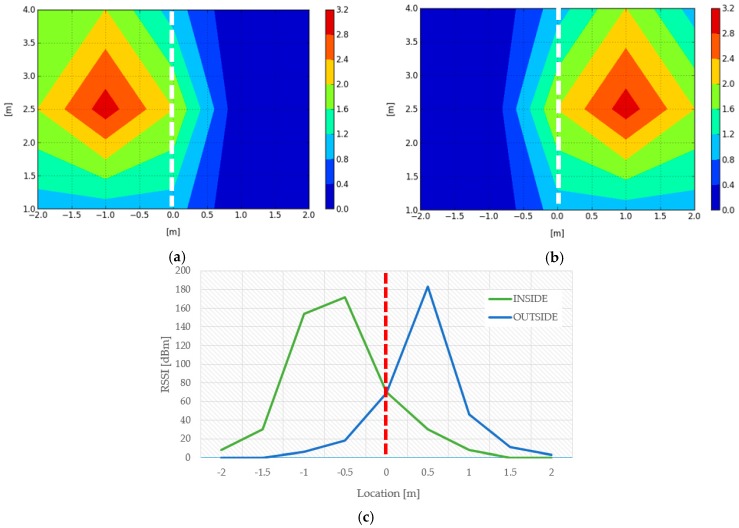
Signal strength between (**a**) inside and (**b**) outside low frequency (LF) transmitters and (**c**) comparison of signal strength.

We also measured the RSSI based on the LF transmitters of the smart box and visualized it to determine the difference in LF RSSI between the inside and outside of the door. Figure 11a shows the signal strength of the inner LF transmitter, Figure 11b shows the signal strength of the outer LF transmitter, and Figure 11c shows the change of the LF RSSI value of the two transmitters according to the variation of the X-axis distance with the Y-axis of Figure 11a,b fixed at 2.5 m. It can be confirmed that the inside and outside are clearly distinguished based on the portion that is near the zero meter (door). Therefore, this allowed for us to monitor mobile assets entering and leaving a 4-meter range of doorway with a pair of LF transmitters. As shown in Figure 11, it can be confirmed that the accuracy of distance recognition by LF-based RSSI guarantees the cm level.

### 5.3. Low Power Consumption in Smart Tag

**Figure 12:**
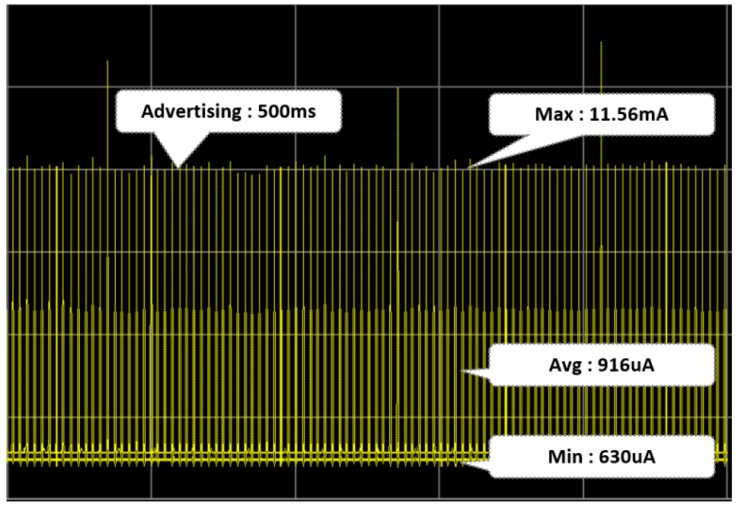
Power status of smart tag (Idle Mode).

The smart tag maintained an idle state while advertising every 500 ms. As shown in Figure 12, when the smart tag was in the idle state, it consumed an average of 916 uA for 1 h. We tested 10 smart tags with a 500-mAh battery, and the smart tag stayed idle for about 20 days. The smart tag was connected to a smartphone via BLE and read each sensor (i.e., temperature, humidity, air pressure, atmosphere, and ultraviolet) data 20 times.

**Figure 13:**
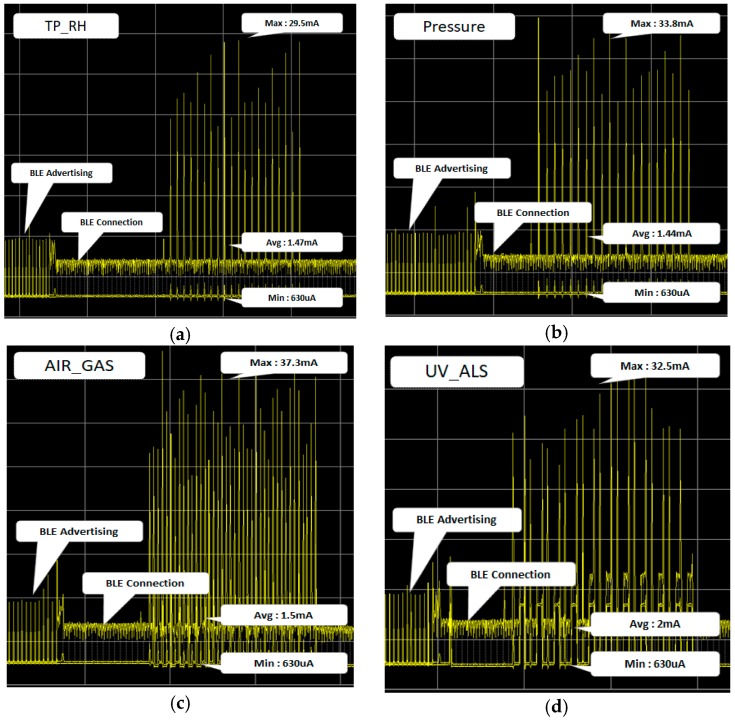
Power status when reading sensors: (**a**) temperature and humidity sensor; (**b**) air pressure sensor; (**c**) atmosphere sensor; (**d**) ultraviolet sensor.

Figure 13 shows the power state of sub-MCU. The base power of sub-MCU did not change, even when reading sensor data. This confirms that the power state of the smart tag did not change, even when sensor data was read.

## 6. Conclusions

In this paper, we proposed the OLCA platform. Unlike existing smart factory technologies, which focus only on production, work environment, and production intelligence, we intended to build an advanced smart factory model by optimizing operator information for each process by implementing a worker-oriented smart factory platform. Additionally, our goal was to construct a platform that could be integrated with existing production facilities and management systems by installing sensing and control devices without changing the existing production processes. We used LAH to provide location-based services, smart boxes to provide opportunistic services, and mobile devices attached to workers and products. LF and BLE communication were used to communicate with each other. Through the modularization of devices, smart factory solutions can be applied while using existing analog factory as-is.

We provide opportunistic, location-based and mobile asset tracking and logging services to the OLCA platform in a factory environment. Since the proposed OLCA platform is a generalized solution that can be used commonly in various types of factories, it can be applied to a wide variety of industries. It also makes it easier to transfer information and data between factories.

In the OLCA platform, opportunistic and location-based services enabled devices to acquire work information on their own, record their work processes and surroundings, and notify operators of problems without intervention. Therefore, we expect to be able to implement a worker-oriented smart factory that communicates with manufacturing facilities and workers directly in the field. 

In order to apply the proposed OLCA model to various factories, it is necessary to understand the equipment characteristics and process characteristics of existing factories. For this purpose, we used an open source CNC machine controller that was provided by GRBL [24]. Anyway, additional work is still needed to connect the existing equipment used at each factory to the OLCA platform, and further research is underway to minimize this work in the future. The OLCA platform will make it easier for even inexperienced workers to handle process systems. It also assumes that the worker’s mistakes will be reduced as a series of work processes are automated. We also pursue that it will become the basis of unmanned smart factory.

## Figures and Tables

**Table 1:** Preparation time of CNC process.

**Preparation Time (s)**	7.7	4.8	4.9	4.9	5.1	4.9	4.9	4.8	6.8	4.8
	4.8	4.9	7.0	4.9	6.8	5.2	6.7	4.7	4.9	4.8
	4.8	4.8	7.1	4.8	6.8	4.8	4.9	4.9	4.8	4.7
	4.8	4.8	4.8	4.8	7.8	5.2	4.8	4.8	4.9	4.7
	4.8	4.8	4.9	4.8	4.9	6.8	5.2	4.9	7.0	4.7
**Average Time (s)**	5.298									
**Standard Deviation** **Time (s)**	0.907									
